# Multimodal neural correlates of dispositional resilience among healthy individuals

**DOI:** 10.1038/s41598-024-60619-0

**Published:** 2024-04-30

**Authors:** Hyun-Ju Kim, Minji Bang, Chongwon Pae, Sang-Hyuk Lee

**Affiliations:** Department of Psychiatry, CHA Bundang Medical Center, CHA University School of Medicine, 59 Yatap-ro, Bundang-gu, Seongnam-si, Gyeonggi-do 463-712 Republic of Korea

**Keywords:** Resilience, Gray matter volume, Local gyrification index, White matter connectivity, Healthy individual, Quality of life, Neuroscience, Psychology

## Abstract

Resilient individuals are less likely to develop psychiatric disorders despite extreme psychological distress. This study investigated the multimodal structural neural correlates of dispositional resilience among healthy individuals. Participants included 92 healthy individuals. The Korean version of the Connor-Davidson Resilience Scale and other psychological measures were used. Gray matter volumes (GMVs), cortical thickness, local gyrification index (LGI), and white matter (WM) microstructures were analyzed using voxel-based morphometry, FreeSurfer, and tract-based spatial statistics, respectively. Higher resilient individuals showed significantly higher GMVs in the inferior frontal gyrus (IFG), increased LGI in the insula, and lower fractional anisotropy values in the superior longitudinal fasciculus II (SLF II). These resilience’s neural correlates were associated with good quality of life in physical functioning or general health and low levels of depression. Therefore, the GMVs in the IFG, LGI in the insula, and WM microstructures in the SLF II can be associated with resilience that contributes to emotional regulation, empathy, and social cognition.

## Introduction

Resilience is a multifaceted construct that can be understood through a range of variables, such as social, ecological, neurobiological, and psychological factors^1–3^. Socio-ecological resilience is defined as the extent to which a specific relationship between social processes and ecological dynamics can be distributed without a significant loss in the complexity of both systems^4^. From a neurobiological perspective, resilience is defined as a dynamic process influenced by neural and psychological self-organization as well as interactions between the ecological context and the developing organism^5^. Much research has been conducted on what was initially revealed phenomenologically regarding psychological resilience. Phenomenological studies have primarily conceptualized psychological resilience as an individual’s ability to adapt despite significant life stressors^6^. Nonetheless, more recent research from a neuroscientific perspective is required to understand the innate mechanisms and processes underlying the phenomenology of resilience^7^. Recent research on resilience has focused on neurobiological mechanisms to understand the complex links between genetic endowments and the environmental impact of resilience^8,9^. Hence, it is essential to understand the role of the neurobiological underpinnings of resilience, because there are multiple environmental factors.

The American Psychological Association defines psychological resilience as the process and outcome of successful adaptation to challenging life experiences such as psychological trauma^10^. Meanwhile, some researchers have suggested that dispositional resilience can be defined more narrowly as an internal resilience factor through individuals’ dispositional attributes (i.e., temperament or personality traits; trait resilience)^11^. Dispositional resilience can be understood as a combination of commitment (engagement with others rather than isolation), control (influence over overcoming rather than feeling powerless), and challenge (learning from experience rather than avoiding threats)^12^. Therefore, investigating the role of dispositional resilience in relation to other resilience factors is essential. High trait resilient individuals exhibit increased neurophysiological and emotional recovery from stressful experiences^13^. The higher the level of dispositional resilience, the lower the level of neuroticism, such as negativity affect-related traits^13^. Dispositional resilience is associated with fewer depressive or anxiety symptoms^14^. Individuals who scored higher on dispositional resilience had more adaptive coping strategies^15^ (i.e., higher problem-solving and lower distance) and higher life satisfaction^16^.

High resilience is closely associated with enhanced social cognition^17^, empathy^18^, emotional regulation^19^, and mindfulness^20^. Resilience facilitates advanced psychological processes, such as effective problem-solving and communication, integral to social behavior^21^. Individuals with higher resilience are better at empathizing with and managing emotions, including the ability to recognize and regulate negative emotions such as fear, anger, and depression^11,22^. Mindfulness also plays a vital role in predicting and enhancing resilience.

Recently, several studies have examined the biological underpinnings of psychological resilience. Previous magnetic resonance image (MRI) based neuroimaging studies using structural MRI (sMRI), diffusion MRI (dMRI), or functional MRI (fMRI) modalities have been conducted to identify neural markers associated with resilience^23^. Some sMRI studies revealed increased gray matter volumes (GMVs) in the prefrontal cortex, anterior cingulate cortex, and amygdala in high-resilient individuals, compared with low-resilient individuals^24,25^. Beyond studies using GMVs, little is known about the differences in resilience that may be found in cortical thickness (CT) or local gyrification index (LGI)—other features of brain structure. Monozygotic twins exhibited variations in gyrification patterns, indicating a more substantial influence of environmental factors on gyrification than on GMVs^26,27^. Additionally, CT and LGI have been used to investigate abnormalities in cortical folding related to trait anxiety^28^. Therefore, differences in gyrification or CT patterns may arise across a larger development window than previously thought. Recent studies have discussed the benefits of combining the two analysis methods^29,30^. In addition, dMRI studies showed that resilient individuals revealed higher fractional anisotropy (FA) values in the anterior corpus callosum and lower FA values in the superior longitudinal fasciculus (SLF) compared to vulnerable individuals^31,32^. However, the studies regarding resilience using sMRI or dMRI may be inconsistent and have difficulty explaining the resilience-related neural correlates by linking them together. Therefore, approaches integrating MRI and dMRI data are needed to examine the neural correlates of dispositional resilience in healthy individuals.

Meanwhile, previous fMRI studies suggested that the connections in a mirror neuron system (MNS) and default mode network (DMN) may be related to psychological resilience. The resilient individuals showed stronger inferior parietal lobe connectivity with the precuneus when viewing happy faces compared to the vulnerable people^33^. In addition, an fMRI study using a task-based approach found that the more resilient individuals who performed the emotion-processing tasks better activated the orbital frontal gyrus and insula^34^. The MNS-related regions, which may contain the inferior parietal lobule, superior temporal sulcus, amygdala, insula, and inferior frontal gyrus (IFG) regions, have been associated with imitation and empathy, which are essential elements for social cognition^35^. The DMN, which may include the medial prefrontal cortex, precuneus, and SLF, is activated during a self-referential task or a relaxed non-task state^36^.

Given neural regions of resilience-related factors (e.g., social cognition, empathy, emotional regulation, and mindfulness), the two major neural regions in humans include a MNS and DMN^37,38^. Particularly, the SLF might be associated with functional connectivity correlates of the DMN^39^ and the FA values of the precuneus white matter (WM), which is a significant region of the DMN and has been found to be negatively correlated with subjective well-being^40^. Furthermore, the studies on dispositional resilience’s neural correlates suggested that they could be utilized for the early detection of low-resilient individuals and applied to neuromodulatory interventions to enhance their resiliency^41–43^.

Therefore, the present study simultaneously examined the structural neural correlates of dispositional resilience in both the gray matter (GM) and WM regions among healthy individuals. It was hypothesized that: (1) there would be an association between dispositional resilience and GMVs, CT, LGI, and WM microstructures in MNS- and DMN-related brain regions; and (2) there would be associations between dispositional resilience’s neural correlates (e.g., GMVs, CT, LGI, and WM microstructures) and other psychological measurements (e.g., coping strategies, trait anxiety, anxiety or depressive symptomatology, and quality of life) among healthy individuals. Therefore, whole-brain analysis of GM and WM using T1 and diffusion tensor imaging (DTI) was performed to detect the structural neural correlates of dispositional resilience among healthy individuals. And then, we performed Spearman’s correlation analyses between the neural correlates of resilience and psychological scales in healthy individuals.

## Results

### Sociodemographics and clinical characteristics

Table 1 displays the characteristics of the 92 participants. The minimum, maximum, mean, and median values of the K-CD-RISC were 42.0, 96.0, 65.1, and 63.0, respectively. According to a standardization study (*N* = 576, at test/*N* = 376, retest)^44^ conducted in healthy Koreans, the mean value (± SD) of K-CD-RISC was 61.2 (± 13.0) at test and 59.3 (± 12.6) at retest. Therefore, according to standardization studies conducted in South Korea, the dispositional resilience scores of the participants included in our study were comparable to Korean norms.

**Table 1:** Sociodemographic and clinical characteristics of healthy individuals (*N* = 92).

Sociodemographic variables	*N* (%) or mean (± SD)
Gender: men/women	43 (46.74)/49 (53.26)
Age at scan (years)	34.32 (± 8.72)
Education year (years)	17.14 (± 2.39)
Intracranial volume (mL)	1529.53 (± 133.76)
Religion: existed/none	37 (40.22)/36 (39.13)
Marital status: living with partner/without partner	30 (32.61)/49 (53.26)
Monthly income: ≥ 1800 $USD/< 1800 $USD	69 (75.0)/3 (3.26)

Clinical characteristics	Mean (± SD) [min–max]
Dispositional resilience (K-CD-RISC total score)	65.10 (± 10.40) [42, 96]
1. Hardiness	22.59 (± 4.61) [11, 36]
2. Persistence	21.10 (± 4.34) [12, 32]
3. Optimism	10.37 (± 2.39) [6, 16]
4. Social support	5.59 (± 1.19) [2, 8]
5. Spiritual influence	4.10 (± 1.47) [1, 7]
Coping strategies (WCQ)	
1. Problem-focused	43.99 (± 1.65) [1, 75]
2. Emotion-focused	11.79 (± 0.71) [2, 33]
Trait anxiety (STAI-trait anxiety score)	35.75 (± 0.89) [23, 55]
Anxiety (BAI total score)	2.55 (± 3.53) [0, 18]
Depression (BDI-II total score)	4.28 (± 4.76) [0, 26]
Life satisfaction (SF-36 total score)	2500.59 (± 389.53) [1495, 3325]
1. Physical functioning	84.86 (± 12.36) [55, 100]
2. Role-physical	73.94 (± 36.95) [0, 100]
3. Role-emotional	74.65 (± 35.40) [0, 100]
4. Energy/fatigue	44.06 (± 11.06) [25, 80]
5. Emotional well-being	56.90 (± 9.05) [40, 80]
6. Social functioning	70.42 (± 13.89) [37.5, 100]
7. Bodily pain	86.44 (± 17.97) [12.5, 100]
8. General health	62.39 (± 15.23) [20, 100]

Values represent count (percent). *SD* standard deviation, *N* number of participants, *min* minimum, *max* maximum, *K-CD-RISC* Korean version of Connor-Davidson Resilience Scale, *WCQ* Way of Coping Questionnaire, *STAI* Spielberger State-Trait Anxiety Inventory, *BDI-II* Beck Depression Inventory-II, *BAI* Beck Anxiety Inventory, *SF-36* Short Form Health Survey-36, *Role-physical* role limitations due to physical health, *Role-emotional* role limitations due to emotional problems.

There were no differences in dispositional resilience scores according to categorical variables, such as gender, presence of religion, marital status, and monthly income. Additionally, no significant correlations were found between dispositional resilience scores, age, and education level.

### Voxel-wise correlation analyses between resilience (K-CD-RISC) and gray matter volume

**Figure 1:**
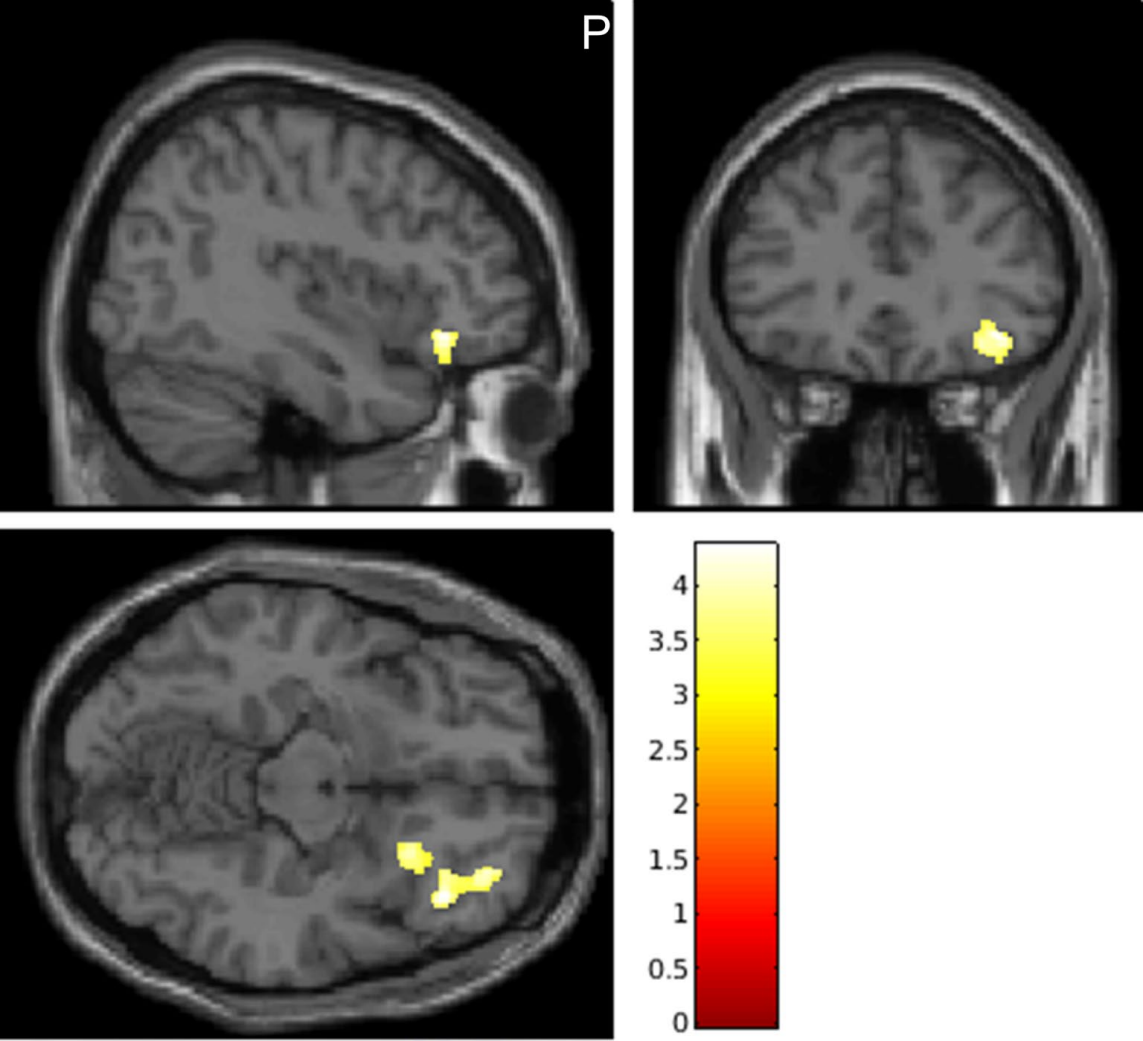
Gray matter neural correlates of resilience among healthy individuals. The gray matter volumes in the inferior frontal gyrus (peak *t*-value = 4.37, cluster size = 664 voxels, MNI coordinate *x* = 38, *y* = 37, *z* = − 15) showed significantly positive correlations with the K-CD-RISC total scores as shown in yellow (voxel threshold: FWE-corrected *p* < 0.05, *k* > 100 voxel). Note: Images of the sagittal (left upper), coronal (right upper), and transversal (left lower) view shown. The color bar shows cluster-level FWE-corrected *p*. *MNI* Montreal Neurological Institute, *K-CD-RISC* Korean version of Connor-Davidson Resilience Scale, *FWE* family-wise error, *p p*-value.

The GMVs in the IFG of the right hemisphere showed a significant positive correlation with the total Korean version of the Connor-Davidson Resilience Scale (K-CD-RISC) scores (FWE-corrected *p* < 0.05) (Fig. 1). The significance was maintained after adjusting for age, gender, and intracranial volume (ICV). However, our study found no significant correlations between the GMVs in the whole brain and K-CD-RISC subscale scores.

### Correlation analyses between resilience (K-CD-RISC), cortical thickness, and local gyrification index

**Figure 2:**
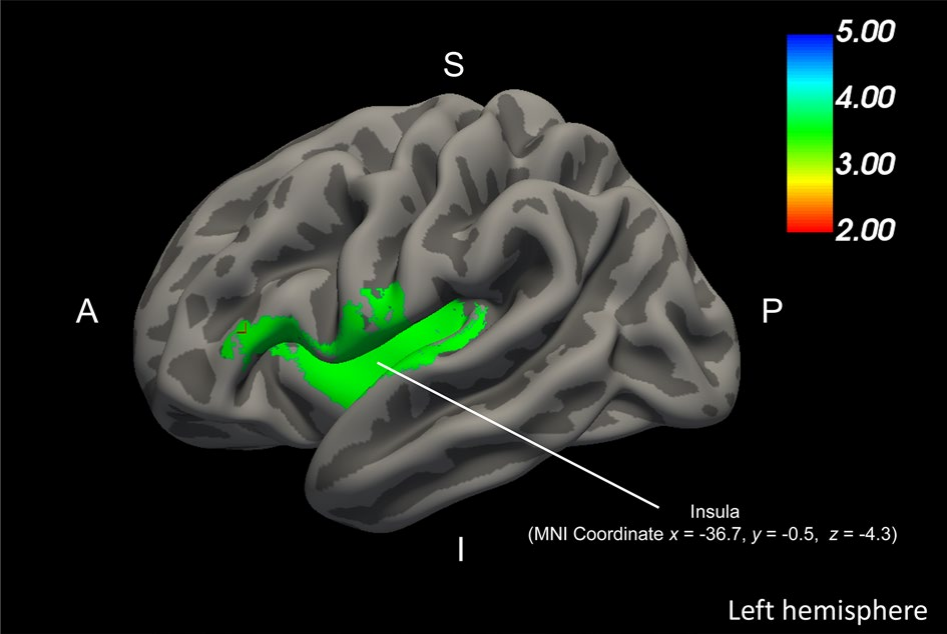
Gray matter neural correlates of resilience among healthy individuals. The local gyrification index in the insula significantly positively correlated with the K-CD-RISC total scores (CWP < 0.05). Note: The color bar shows cluster-level FWE-corrected *p*. *K-CD-RISC* Korean version of Connor-Davidson Resilience Scale, *p p*-value, *MNI* Montreal Neurological Institute, *CWP* cluster wise *p*-value.

Concerning the LGI, the insula in the left hemisphere showed a significant positive correlation with the total K-CD-RISC scores (Fig. 2). The relationships between the K-CD-RISC subscale scores and the LGIs in the whole brain have been illustrated in Supplementary Fig. 1. However, no significant correlations were found between healthy individuals’ total or subscale scores of K-CD-RISC and CT in the whole brain.

### Voxel-wise correlation analyses between the resilience (K-CD-RISC) and the mean fractional anisotropy values

**Figure 3:**
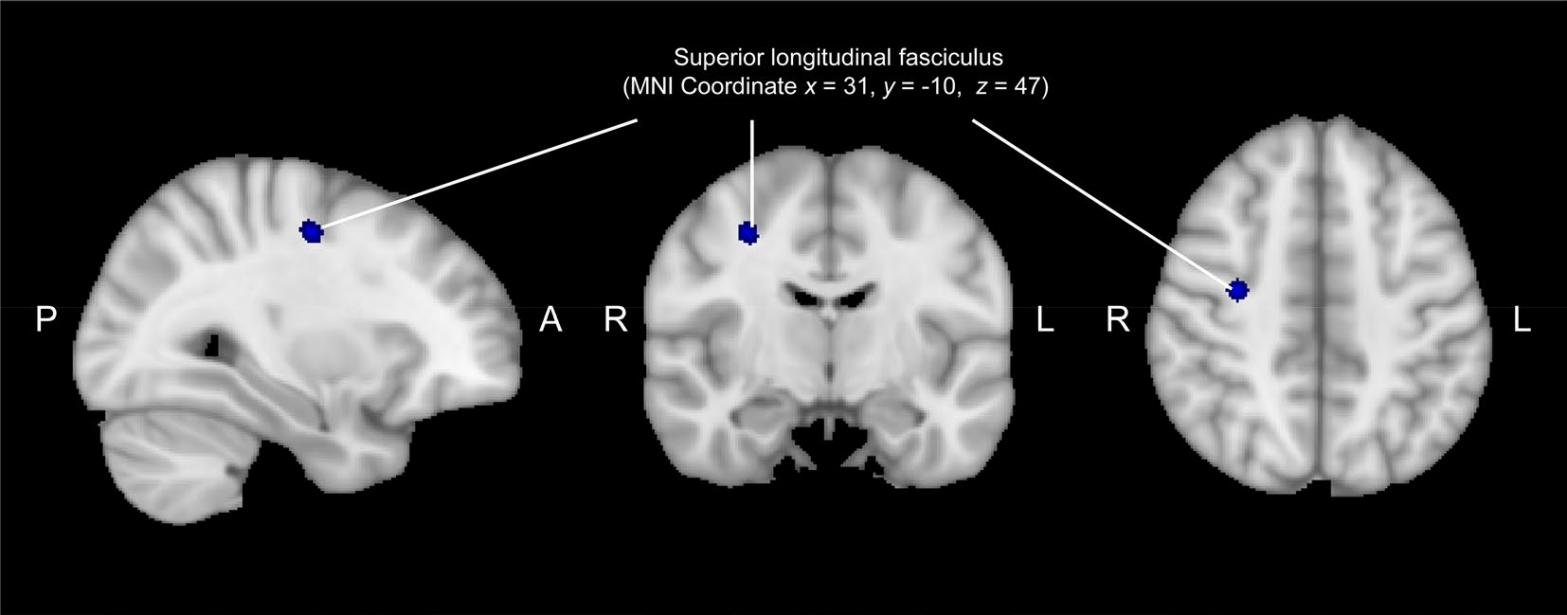
White matter neural correlates of resilience among healthy individuals. The mean fractional anisotropy values of the second branch of superior longitudinal fasciculus showed a significantly negative correlation with the K-CD-RISC total scores as shown in blue (voxel threshold: TFCE-corrected *p* < 0.05). Note: Images of the sagittal, coronal, and transversal view shown overlaid on the MNI 1 mm template. *MNI* Montreal Neurological Institute, *K-CD-RISC* Korean version of Connor-Davidson Resilience Scale, *TFCE* threshold-free cluster enhancement.

Figure 3 illustrates the voxel-wise correlation analyses between the total K-CD-RISC scores and the mean FA values of the whole brain among healthy individuals. Healthy individuals with higher total K-CD-RISC scores showed significantly lower mean FA values in the second branch of the SLF (SLF II) (TFCE-corrected *p* < 0.05). Significance was maintained even after adjusting for age and gender as covariates. The relationships between the subscale scores of the K-CD-RISC and the mean FA values of WM have been shown in Supplementary Fig. 2.

### Spearman’s correlation analyses between the structural neural correlates of resilience (GMVs of the IFG, LGI in the Insula, and mean FA of the SLF II) and other psychological measurements

**Figure 4:**
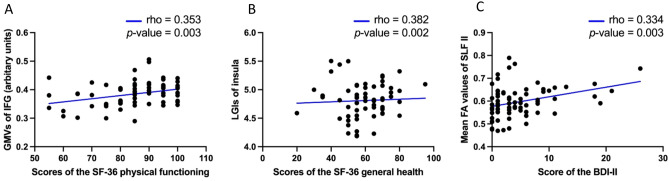
Results of the Spearman’s correlation analyses after Bonferroni correction for multiple comparisons showed significant positive correlations between the structural neural correlates of the resilience [(**A**) GMVs of IFG, (**B**) LGIs of the insula, and (**C**) mean FA values of SLF II] and other psychological measures among healthy individuals. *GMVs* gray matter volumes, *IFG* inferior frontal gyrus, *LGI* local gyrification index, *FA* fractional anisotropy, *SLF II* the second branch of the superior longitudinal fasciculus, *SF-36* Short Form health survey-36, *BDI-II* Beck Depression Inventory-II.

Figure 4 shows Spearman’s correlation analysis findings after Bonferroni correction for multiple comparisons between the structural neural correlates of resilience (K-CD-RISC total scores) and psychological measurements among healthy individuals (Fig. 4). Figure 4A shows the association between the IFG’s GMVs and the 36-item Short Form Health Survey (SF-36) physical functioning scores (*rho* = 0.353, *p*-value = 0.003). The insula LGIs significantly positively correlated with the SF-36 general health scores (*rho* = 0.382, *p*-value = 0.002, Fig. 4B2). In Fig. 4C, the mean FA values of SLF II were significantly positively correlated with Beck Depression Inventory-II (BDI-II) total scores (*rho* = 0.334, *p*-value = 0.003).

Additionally, we presented Spearman’s correlation analysis findings that did not survive correction for multiple comparisons (Supplementary Fig. 3). The LGIs of the insula negatively correlated with Beck Anxiety Inventory (BAI) total scores (*rho* = − 0.347, *p*-value = 0.005). The mean FA values of SLF II were positively associated with the Spielberger State-Trait Anxiety Inventory (STAI)-trait anxiety total scores (*rho* = 0.263, *p*-value = 0.030) and BAI total scores (*rho* = 0.258, *p*-value = 0.030). Furthermore, the coping strategy measurements were not significantly correlated with the structural neural correlates of resilience among healthy individuals.

The results of Spearman’s correlation analyses between the structural neural correlates of the resilience subscales and psychological assessments among healthy individuals have been illustrated in Supplementary Fig. 4.

## Discussion

This study marks a significant advance in neuroimaging studies of resilience, as it is the first to uncover specific neuroanatomical markers in healthy individuals with high resilience, a focus not previously explored. We hypothesized notable associations between dispositional resilience and neuroanatomical features, particularly in the GMV, CT, LGI, and WM microstructures within the MNS and DMN regions. Additionally, we expected to identify correlations between these neural markers and several psychological parameters in healthy individuals. Our results confirm these hypotheses, showing that high resilience is associated with increased GMVs in the IFG regions, increased LGI in the insula, and reduced FA in the SLF II. This adds a new dimension to our understanding of the relationship between resilience and brain structure. These structural brain differences are consistent with our predictions regarding the neuroanatomical basis of resilience and are correlated with psychological well-being. Specifically, we observed that increased GMVs in the IFG were positively associated with dispositional resilience and physical functioning, suggesting a link between this brain region and overall health. Similarly, increased LGI in the insula correlated with lower levels of anxiety, and changes in the WM microstructure in the SLF II were associated with reduced depression and anxiety, further supporting the link between resilience, brain structure, and psychological states.

This study found a significant positive association between resilience and GMVs in the IFG. A previous resting-state fMRI study of healthy individuals showed that the resilient group had significantly greater IFG activation^45^. Voxel-based morphology analyses revealed that GMVs in the IFG and insula significantly increased in the relatively high resilience group compared to the middle-level resilience group^46^. These studies underscore the IFG and insula as regions directly or indirectly related to pain resilience processing and are indirectly linked to ongoing adversity. These results were consistent with our research findings. The IFG plays a significant role in emotional empathy, emotion recognition, working memory, inhibition, and attentional control^47–49^. Particularly, the human IFG is situated in a virtually mirror-like location for processing empathy and is associated with the MNS. Several functional neuroimaging studies have shown that neurons in the IFG may be associated with the imitation of movement, observation, and nonverbal communication in social cognition^50–52^. More resilient individuals showed more appropriate emotional responses in social contexts. This may be associated with their high social competence, which in turn might be associated with neural circuits such as the MNS. Among MNS-related domains, IFG cognitively regulates emotions and may have a significant association with the K-CD-RISC, which includes hardiness. Therefore, the increased GMVs in the IFG in the present study may indicate that highly resilient healthy individuals respond with appropriate cognitive-emotional control in a social context, although the causal relationship is unknown.

Our study showed that GMVs in the IFG were positively correlated with physical functioning among healthy individuals. The IFG is crucial for the processing and organization of actions during behaviors^53^. The ventral region of the right IFG played a crucial role in updating the corresponding action plan. A previous study has demonstrated a correlation between greater GMVs in the IFG and physical activity among healthy adults^54^. These findings are consistent with our results.

Our finding showed a significant positive correlation between psychological resilience and the insula’s LGIs and their link to a high quality of life in general health and low anxiety levels. This supports our hypothesis that resilience might be associated with the MNS- or DMN-related brain regions of social cognition or empathy. Especially, the insula integrates information from cognitive, emotional, and affective processes^55^. As a vital region in the salience network, the insula integrates information from cognitive, emotional, and affective processes^55–58^. The insula-anterior cingulate cortex (ACC) circuitry may be crucial in stress adaptation mechanisms underpinned by the interplay between the DMN and salience networks^59^. Based on previous studies, the insula seems to contribute to an individual’s emotional regulation and increased optimism, ultimately enhancing resilience^60^. A previous study found that the local activation of the insula and ACC was negatively correlated with resilience in a healthy population^61^. In addition, Waugh et al.^62^ have showed that in face of fear, high dispositional resilient healthy individuals show insula activation only to the aversive pictures, suggesting that resilient people can flexibly use emotional resources, whereas low-resilient individuals exhibit insula activation to both the aversive and neutral pictures. Thus, in this context, the increased LGIs in the insula among highly more resilient people may be relatively better at regulating their cognitive and emotional process, and this may ultimately be related to improving their overall quality of life.

Our study showed that highly resilient healthy individuals had significantly lower FA values in the SLF II. Studies on WM connectivity in psychological resilience have shown inconsistent results; in some reports, significant positive correlations were seen between psychological resilience and WM connectivity within the corpus callosum, SLF, superior corona radiata, or internal capsule^31,32^ while the opposite effect has been noted in others^63^. Especially, the SLF is a significant association fiber encompassing the frontal, parietal, and temporal areas; it plays an instrumental role in cognitive function^64,65^. The SLF II is a major component of the SLF and central fibers of the WM located above the insula; it anatomically connects the angular gyrus and parietal lobe with the IFG in humans^66^. SLF II generally involves visuospatial awareness and executive functions such as working memory^67,68^. Besides these functions, the SLF II is essential in regulating spatial attention because it consists of DMN nodes^69–71^. A previous neuroimaging study among healthy individuals has shown that the decreased mean FA values in the DMN-related SLF regions might be associated with creative cognition, which is similar to the finding of decreased activation in DMNs after meditation practice^72,73^. Another previous study has demonstrated a positive relationship between FA values and functional connectivity in the DMN of healthy individuals^74^. Therefore, the reduced FA values in the SLF II among healthy individuals in our study might be associated with divergent thinking ability and a stable meditative state, which may be associated with higher adaptive and resilient capacities.

Moreover, Spearman’s correlation analyses revealed that higher dispositional resilience-associated negative FA values in the SLF II were associated with lower levels of traits, state anxiety, and depression. Previous fMRI studies have emphasized the importance of dispositional resilience in emotional regulation in healthy individuals^60,75–77^. In particular, longitudinal fMRI studies of resilience have found that lower engagement of response areas of the threat network and DMN, including the amygdala and posterior cingulate cortex regions, may have a positive effect on resilience shortly after experiencing psychological trauma^77^. Given this neuroimaging finding, the lower WM connectivity of the SLF, which are DMN-related emotional regulation regions^78,79^, might appear to be protective against psychological distress in healthy individuals in our study. Lower FA values in SLF II in healthy individuals may be associated with positive personal dispositions or attitudes, such as self-compassion^80^. One study reported that an increase in the FA of the uncinate fasciculus was negatively associated with psychological resilience as measured by the presence of more anxiety-related symptoms^81^. Another neuroimaging study suggested that lower WM microstructural integrity in the uncinate fasciculus, which is considered a part of the limbic system, may represent a lower negative affect trait in healthy adult individuals^82^. Based on these findings, the current results indicate that lower WM connections in SLF II, which could be structural neural correlates of high dispositional resilience, may be related to lower negative emotions and affect.

In terms of examining the neural correlates of the resilience subscales, we found that LGIs in the superior temporal and supramarginal gyri were significantly positively associated with *hardiness* scores among healthy individuals. Additionally, the LGIs in the insula and rostral middle frontal gyrus were significantly positively correlated with *persistence* scores. We also found significant negative associations between mean FA values in the three cluster regions (SLF II, inferior longitudinal fasciculus, and posterior WM) and *persistence* scores. Our findings indicated that the persistence subscale was most strongly associated with the total resilience scale among healthy individuals. Previous research suggests that resilience-related psychological constructs in healthy individuals include confidence, self-control, persistence^83^, optimism^84^, agreeableness^85^, and emotional stability^86^. Therefore, it is difficult to determine the most important factors affecting resilience. However, we inferred from our findings that “*persistence*” may be a crucial factor in dispositional resilience.

Our study has several limitations. First, dispositional resilience was assessed using a validated self-report K-CD-RISC scale, which could be influenced by reporting bias. Second, this was a cross-sectional study with participants who might have experienced stressful or negative life events and were currently healthy. Additional longitudinal studies may be useful for evaluating the effects of exposure to stressful life events on the association between changes in the brain and dispositional resilience. Third, the current study conducted a structural brain imaging analysis to examine the neural correlates of dispositional resilience and their relationships with psychological constructs among healthy individuals, instead of using functional brain imaging. Although structural brain imaging provides valuable insights into the underlying anatomy of the brain, it is also important to investigate the dynamic process of positive adaptation that characterizes resilience within the context of adversity. Therefore, it would be meaningful to investigate changes in resilience in brain regions using resting-state or task-based brain activity and connectivity, as well as to explore their associations with other psychological measurements using functional imaging analysis in the future^87,88^.

In conclusion, our findings indicate that the increased GMVs or LGI in the social cognition-related IFG or insula and decreased WM microstructures in the DMN-related regions, including SLF II, were associated with high resilience among healthy individuals. Our study suggests that alterations of neural correlates in highly resilient individuals might be associated with lower psychological distress and higher quality of life, particularly in terms of physical functioning or general health. Therefore, neuronal changes in these dispositional resilience-related brain structures may be associated with the capacity to overcome stressful life events and achieve a high quality of life among healthy individuals.

## Materials and methods

### Participants

Ninety-two right-handed healthy individuals (43 men and 49 women, mean age = 34.32 ± 8.72 years) from the local community of Seongnam City were recruited through advertisements to participate in this study. All participants completed the resilience assessment and MRI. The exclusion criteria were: (1) any history of major psychiatric disorders, including schizophrenia spectrum disorders, depressive disorders, bipolar disorders, anxiety disorders, and substance use disorders; (2) neurological disorders and traumatic brain injury; (3) pregnancy; (4) any contraindication for MRI scanning; and (5) no history of mental illness in first-degree relatives.

The recommendations and ethics of the Institutional Review Board of the CHA Bundang Medical Center (2019-05-030) were followed to perform the study procedures. Sufficient information was provided to all participants and their written informed consent was obtained following the latest version of the Declaration of Helsinki and the principles of good clinical practice.

### Dispositional resilience

The K-CD-RISC was used to measure dispositional resilience as an individual’s ability to cope with stress and adversity^89^. This 25-item scale is used for the general healthy population and is rated on a 5-point Likert scale (0 to 4), with total scores ranging from 0 to 100. Higher total K-CD-RISC scores indicate higher psychological dispositional resilience. The K-CD-RISC has five subscales: hardiness, persistence, optimism, social support, and spiritual influence^90^. Each domain comprises eight, eight, five, three, and two items, respectively. Additionally, it has a high Cronbach’s *α* coefficient (0.93) and test–retest reliability (0.93) for measuring dispositional resilience among the South Korean population^90^.

### Other psychological measurements

The Ways of Coping Questionnaire (WCQ) is used to evaluate coping strategies during stressful experiences^91,92^. This 50-item scale is rated on a 5-point Likert scale. Coping styles can be divided into the problem- and emotion-focused coping styles^93^. The Korean version of the WCQ (WCQ-K), including the problem- and emotion-focused coping subscales, was used in this study, with total scores ranging from 0 to 186. The WCQ-K has relatively high internal consistency (Cronbach’s *α* = 0.90) and test–retest reliability (*r* = 0.80)^94^.

The STAI^95,96^ was used to evaluate trait anxiety. The STAI-trait anxiety subscale comprises 20 items that measure anxiety as a personality trait. It is rated on a 4-point Likert scale (1 to 4), and has a high Cronbach’s *α* coefficient (0.89) among the South Korean population^97^. The scale ranges from 20 to 80.

The Korean version of the BAI was used to evaluate the severity of anxiety symptoms. This 21-item self-report inventory evaluates the severity of clinical anxiety and is rated on a 4-point Likert scale ranging from 0 to 3^98^, which ranges from 0 to 63. Cronbach’s *α* coefficient and test–retest reliability for this scale were 0.91 and 0.84, respectively, among the Korean adult population^99^.

Additionally, the Korean version of the BDI-II was used to assess the severity of depressive symptoms. This 21-item self-administered inventory is rated on a 4-point Likert scale (0 to 3), with total scores ranging from 0 to 63^100^. It has high internal consistency, with a Cronbach’s *α* coefficient of 0.93 among healthy adults^101^.

The SF-36 was used to measure health-related quality of life^102^. For the Korean version of the SF-36, Cronbach’s *α* coefficient ranged from 0.93 to 0.94, while the test–retest reliability coefficients ranged from 0.71 to 0.90^103^. The SF-36 is scored differently for each of the 36 items, resulting in a score from 0 to 100 for each item, with a maximum total score of 3600.

### Neuroimaging data acquisition and analyses

#### MRI data acquisition

T1- and diffusion-weighted images (DWIs) were acquired on a 3.0-Tesla MR scanner (GE Healthcare, Milwaukee, WI, USA) with an eight-channel head coil. The parameters of the 3D T1-weighted fast spoiled gradient-echo sequence were: repetition time of 6.3 ms, echo time of 2.1 ms, flip angle of 12°, slice thickness of 1 mm, field of view of 256 × 256 mm^2^, matrix of 256 × 256, and isotropic voxel size of 1 × 1 × 1 mm^3^.

An echo-planar imaging (EPI) sequence was utilized for DWIs (repetition time = 17,000 ms, echo time = 108 ms, field of view = 240 mm, matrix size = 144 × 144, slice thickness = 1.7 mm, and voxel size = 1.67 × 1.67 × 1.7 mm^3^). Eddy current effects were minimized by applying the double-echo option. An eight-channel head coil and an array of spatial sensitivity encoding techniques (GE Healthcare) with two sensitivity encoding speed-up factor were used to reduce the impact of EPI spatial distortions. Seventy axial slices parallel to the anterior–posterior commissure line covering the entire brain in 51 directions with a b-value of 900 s/mm^2^ and eight baseline scans with a b-value of 0 s/mm^2^ were acquired. The DTIs were approximated from DWIs using the least-squares method (approximate scan time: 17 min).

#### VBM image processing and analysis

Voxel-based morphometry (VBM)^104^ was performed using Statistical Parametric Mapping 12 (SPM12, http://www.fil.ion.ucl.ac.uk/spm) and Computational Anatomy Toolbox 12 (CAT12, https://neuro-jena.github.io/cat/) was implemented in MATLAB R2021a. The sensitivity offered by CAT12 is equivalent to that derived from FreeSurfer but with the added advantage of less computational consumption. After reorienting the T1-weighed images to define the anterior commissure as the origin, they were segmented into three parts: GM, WM, and cerebrospinal fluid (CSF). T1-weighted images were normalized into a Montreal Neurologic Institute (MNI) space using CAT12. All normalized and modulated GMVs were smoothed using an isotropic Gaussian kernel with a 6 mm full-width at half-maximum (FWHM). An absolute threshold masking of 0.1 was applied to restrict the voxels of GMVs only.

Whole-brain multiple regression analysis was performed using the total or five subscale scores of K-CD-RISC as an independent variable using CAT12. Additionally, total ICV, age, and gender were adjusted as covariates to control for potential confounding variables. The significance threshold was set at a cluster-level familywise error (FWE)-corrected *p* < 0.05 using the value provided in SPM12. For further correlational analysis, the MarsBar toolbox was used to extract the mean GMVs in a cluster that showed a significant correlation with the total K-CD-RISC scores for each participant^105^.

#### T1-weighted image processing and surface-based analysis

The T1-weighted standard image processing and surface-based analysis were performed using FreeSurfer (version 7.1.0; http://surfer.nmr.mgh.harvard.edu) to estimate the CT and LGI of GM. Our study conducted the quality check based on the ENIGMA QC protocols (https://enigma.ini.usc.edu/protocols/imaging-protocols). All T1-weighted images of participants passed the QC. First, all participants were processed using the fully automated FreeSurfer “*recon-all (-lgi)*” standard procedure. This pipeline includes nonuniform intensity correction, skull stripping, talairach transforms, normalization and atlas registration, subcortical segmentation, surface reconstruction, cortical atlas registration, and segmentation. Second, the CT was calculated as the closest distance from the GM-WM boundary to the GM-CSF boundary at each vertex on the surface^106^. The LGI was measured as the ratio of the local surface area to the outer hull layer that tightly wrapped the pial surface^107^, which is an indication of the sulcal cortex buried in its locality and thus denotes the extent of cortical folding^108^. Finally, the cortical maps of both hemispheres were smoothed with a circularly symmetric Gaussian kernel with 10 mm FWHM to provide a normal distribution of the results. The cortex was auto-parcellated into 34 different gyral regions per hemisphere using the gyral and sulcal anatomy. The mean CT and LGI values were calculated for each of these regions using the Desikan–Killiany atlas as a reference^109,110^.

We conducted a generalized linear regression analysis including the variables resilience and CT and LGI in the whole brain using FreeSurfer command “*mri_glmfit*”. The K-CD-RISC total and five subscale scores were used as independent variables; total ICV, age, and gender were controlled as covariates. Ten-thousand iterations of the Monte Carlo simulation were performed to identify significant clusters between resilience and CT or LGI. The significance threshold was set at cluster-wise probability (CWP) < 0.05. The distribution of the maximum cluster sizes under the null hypothesis was evaluated to correct for multiple comparisons at the cluster level. Peak clusters were identified, and the maximum cluster size was used for correction.

#### DTI image processing and analysis

Voxel-wise statistical analysis of FA data was performed to analyze WM microstructures using the Tract-Based Spatial Statistics (TBSS) version 1.2, implemented in the Functional Magnetic Resonance Imaging of the Brain (FMRIB) Software Library (FSL version 6, Oxford, UK; https://fsl.fmrib.ox.ac.uk/fsl) according to the standard procedure^111^. FA is a measure of the extent of an ellipsoid that provides information regarding the degree of anisotropy in a voxel. Initially, DTI preprocessing, including skull stripping using the brain extraction tool and eddy current correction, was performed using FSL^112^. The FMRIB’s Nonlinear Image Registration Tool was performed to align all participants’ FA data into the standard space (MNI 152 standard). All the transformed FA images were combined and applied to the original FA map to create a standard space version of the FA map. Then, all transformed FA images were averaged to create a mean FA image that was skeletonized to produce a mean FA skeleton regarding the center of the WM regions only. The skeleton’s threshold was set to FA > 0.2 (the default TBSS) to contain only major fiber bundles (vertex-wise threshold of *p* < 0.05)^113^.

Correlation analyses were performed to evaluate whether regional differences in WM integrity were potentially correlated with variance in psychological resilience (K-CD-RISC total or subscale scores). The DTI data were analyzed in the whole brain using the TBSS General Linear Model regression analysis running the command “*randomise*” from FSL. To confirm the voxel-wise statistical analysis findings, the mean FA values were extracted from the skeletonized major fiber tract clusters that demonstrated a significant association with the K-CD-RISC total or five subscale scores. A threshold-free cluster enhancement (TFCE) approach was used to correct for multiple comparisons. The threshold for significance was set at *p*-value < 0.05.

### Additional statistical analyses

Our study conducted a statistical power analysis to estimate the sample size using the G-Power program^114^. We entered the following values: effect size = 0.50 (medium size), two-sided type I error probability α = 0.05, and power = 0.95. Using these parameter values in the program gives a total sample size of *n* = 42, which is needed to detect an actual statistical power of 95.45%.

Correlation analyses were conducted between the K-CD-RISC-related neural correlates (i.e., GMVs, LGI, and mean FA) and psychological clinical scales (WCQ, STAI-trait anxiety, BDI-II, BAI, and SF-36). Spearman’s method was used because the psychological scales utilized in this study were primarily designed for psychiatric patients. The scores of psychological scales did not follow a normal distribution when used with healthy individuals. The Bonferroni correction was applied for multiple comparison tests. Statistical analyses were conducted using SPSS software Version 27.0 (IBM Corp., Armonk, NY, USA).

### Supplementary Information

Supplementary Information.

Supplementary Figures.

## Data Availability

The original contributions presented in the study are included in this article. Further inquiries can be directed to the corresponding authors.

## References

[1] Russo SJ (2012). Neurobiology of resilience. Nat. Neurosci..

[2] Fletcher D, Sarkar M (2013). Psychological resilience. Eur. Psychol..

[3] Ungar M, Theron L (2020). Resilience and mental health: How multisystemic processes contribute to positive outcomes. Lancet Psychiatry.

[4] Goldstein BE (2008). Skunkworks in the embers of the cedar fire: Enhancing resilience in the aftermath of disaster. Hum. Ecol..

[5] Curtis WJ, Cicchetti D (2003). Moving research on resilience into the 21st century: Theoretical and methodological considerations in examining the biological contributors to resilience. Dev. Psychopathol..

[6] Masten AS, Gordon E, Wang M (1994). Educational Resilience in Inner City America: Challenges and Prospects, Ch. 3.

[7] Richardson GE (2002). The metatheory of resilience and resiliency. J. Clin. Psychol..

[8] Kalisch R (2015). A conceptual framework for the neurobiological study of resilience. Behav. Brain Sci..

[9] Rutten BP (2013). Resilience in mental health: Linking psychological and neurobiological perspectives. Acta Psychiatr. Scand..

[10] Newman R (2005). APA’s resilience initiative. Prof. Psychol. Res. Pract..

[11] Wolin S, Wolin S (1993). Bound and Determined: Growing Up Resilient in a Troubled Family.

[12] Antonovsky, A. Health, stress, and coping. In *New Perspectives on Mental and Physical Well-being* 12–37 (1979).

[13] Fredrickson BL (2003). What good are positive emotions in crisis? A prospective study of resilience and emotions following the terrorist attacks on the United States on September 11th, 2001. J. Pers. Soc. Psychol..

[14] Steinhardt M, Dolbier C (2008). Evaluation of a resilience intervention to enhance coping strategies and protective factors and decrease symptomatology. J. Am. Coll. Health.

[15] Dumont M, Provost MA (1999). Resilience in adolescents: Protective role of social support, coping strategies, self-esteem, and social activities on experience of stress and depression. J. Youth Adolesc..

[16] Abolghasemi A, Varaniyab ST (2010). Resilience and perceived stress: Predictors of life satisfaction in the students of success and failure. Procedia Soc. Behav. Sci..

[17] Garmezy N, Masten AS (1986). Stress, competence, and resilience: Common frontiers for therapist and psychopathologist. Behav. Ther..

[18] Werner, E. E. Stress and protective factors in children’s lives. *Child Psychol. Psychiatry* 335–355 (1985).

[19] Kumpfer KL, Johnson JL, Glantz MD (2002). Resilience and Development.

[20] Keye MD, Pidgeon AM (2013). Investigation of the relationship between resilience, mindfulness, and academic self-efficacy. Open J. Soc. Sci..

[21] Melloni M (2014). Empathy and contextual social cognition. Cogn. Affect. Behav. Neurosci..

[22] Troy AS, Mauss IB, Litz BT, Southwick SM, Charney D, Friedman MJ (2011). Resilience and Mental Health: Challenges Across the Lifespan.

[23] Eaton S (2022). Resilience and young people’s brain structure, function and connectivity: A systematic review. Neurosci. Biobehav. Rev..

[24] Burt KB (2016). Structural brain correlates of adolescent resilience. J. Child Psychol. Psychiatry.

[25] Morey RA (2016). Amygdala, hippocampus, and ventral medial prefrontal cortex volumes differ in maltreated youth with and without chronic posttraumatic stress disorder. Neuropsychopharmacology.

[26] White T (2002). Brain volumes and surface morphology in monozygotic twins. Cereb. Cortex.

[27] Bartley AJ (1997). Genetic variability of human brain size and cortical gyral patterns. Brain.

[28] Miskovich TA (2016). Cortical gyrification patterns associated with trait anxiety. PLoS One.

[29] Winkler AM (2010). Cortical thickness or grey matter volume? The importance of selecting the phenotype for imaging genetics studies. NeuroImage.

[30] Goto M (2022). Advantages of using both voxel- and surface-based morphometry in cortical morphology analysis: A review of various applications. Magn. Reson. Med. Sci..

[31] Galinowski A (2015). Resilience and corpus callosum microstructure in adolescence. Psychol. Med..

[32] Jones SA (2019). Resilience to risk for psychopathology: The role of white matter microstructural development in adolescence. Biol. Psychiatry Cogn. Neurosci. Neuroimaging.

[33] Nimarko AF (2019). Neural correlates of emotion processing predict resilience in youth at familial risk for mood disorders. Dev. Psychopathol..

[34] Heitzeg MM (2008). Affective circuitry and risk for alcoholism in late adolescence: Differences in frontostriatal responses between vulnerable and resilient children of alcoholic parents. Alcohol. Clin. Exp. Res..

[35] Iacoboni M, Dapretto M (2006). The mirror neuron system and the consequences of its dysfunction. Nat. Rev. Neurosci..

[36] Raichle ME (2015). The brain’s default mode network. Annu. Rev. Neurosci..

[37] Rizzolatti G, Fabbri-Destro M (2008). The mirror system and its role in social cognition. Curr. Opin. Neurobiol..

[38] Mars RB (2012). On the relationship between the “default mode network” and the “social brain”. Front. Hum. Neurosci..

[39] Pisner D (2019). The superior longitudinal fasciculus and its functional triple-network mechanisms in brooding. Neuroimage Clin..

[40] Jung H-Y (2022). A multimodal study regarding neural correlates of the subjective well-being in healthy individuals. Sci. Rep..

[41] Kahl M (2020). Resilience and cortical thickness: A MRI study. Eur. Arch. Psychiatry Clin. Neurosci..

[42] Jeong H (2021). Increased medial prefrontal cortical thickness and resilience to traumatic experiences in North Korean refugees. Sci. Rep..

[43] Salehinejad MA (2017). Neural correlates of trait resiliency: Evidence from electrical stimulation of the dorsolateral prefrontal cortex (dLPFC) and orbitofrontal cortex (OFC). Pers. Individ. Differ..

[44] Baek H-S (2010). Reliability and validity of the Korean version of the Connor–Davidson Resilience Scale. Psychiatry Investig..

[45] Santarnecchi E (2015). The smarter, the stronger: Intelligence level correlates with brain resilience to systematic insults. Cortex.

[46] Li F, Jackson T (2020). Gray matter volume differences between lower, average, and higher pain resilience subgroups. Psychophysiology.

[47] Hampshire A (2010). The role of the right inferior frontal gyrus: Inhibition and attentional control. Neuroimage.

[48] Liakakis G (2011). Diversity of the inferior frontal gyrus—A meta-analysis of neuroimaging studies. Behav. Brain Res..

[49] Shamay-Tsoory SG (2009). Two systems for empathy: A double dissociation between emotional and cognitive empathy in inferior frontal gyrus versus ventromedial prefrontal lesions. Brain.

[50] Schulte-Rüther M (2007). Mirror neuron and theory of mind mechanisms involved in face-to-face interactions: A functional magnetic resonance imaging approach to empathy. J. Cogn. Neurosci..

[51] Newman-Norlund RD (2007). The mirror neuron system is more active during complementary compared with imitative action. Nat. Neurosci..

[52] Cheng Y (2007). Motivation modulates the activity of the human mirror-neuron system. Cereb. Cortex.

[53] Binkofski F, Buccino G (2006). The role of ventral premotor cortex in action execution and action understanding. J. Physiol. Paris.

[54] Erickson KI (2010). Physical activity predicts gray matter volume in late adulthood: The Cardiovascular Health Study. Neurology.

[55] Li F (2020). Neuroanatomical and functional alterations of insula in mild traumatic brain injury patients at the acute stage. Brain Imaging Behav..

[56] Menon V, Uddin LQ (2010). Saliency, switching, attention and control: A network model of insula function. Brain Struct. Funct..

[57] Gasquoine PG (2014). Contributions of the insula to cognition and emotion. Neuropsychol. Rev..

[58] Picó-Pérez M (2019). Common and distinct neural correlates of fear extinction and cognitive reappraisal: A meta-analysis of fMRI studies. Neurosci. Biobehav. Rev..

[59] Shao R (2018). Subgenual anterior cingulate-insula resting-state connectivity as a neural correlate to trait and state stress resilience. Brain Cogn..

[60] Tai AP (2023). Conceptualizing psychological resilience through resting-state functional MRI in a mentally healthy population: A systematic review. Front. Behav. Neurosci..

[61] Kong F (2015). Neural correlates of psychological resilience and their relation to life satisfaction in a sample of healthy young adults. Neuroimage.

[62] Waugh CE (2008). The neural correlates of trait resilience when anticipating and recovering from threat. Soc. Cogn. Affect. Neurosci..

[63] Anacker C (2016). Neuroanatomic differences associated with stress susceptibility and resilience. Biol. Psychiatry.

[64] Petrides M, Pandya DN (1984). Projections to the frontal cortex from the posterior parietal region in the rhesus monkey. J. Comp. Neurol..

[65] Nakajima R (2020). The superior longitudinal fascicle: Reconsidering the fronto-parietal neural network based on anatomy and function. Brain Imaging Behav..

[66] Makris N (2005). Segmentation of subcomponents within the superior longitudinal fascicle in humans: A quantitative, in vivo, DT-MRI study. Cereb. Cortex.

[67] Nakajima R (2017). Damage of the right dorsal superior longitudinal fascicle by awake surgery for glioma causes persistent visuospatial dysfunction. Sci. Rep..

[68] Curtis CE (2006). Prefrontal and parietal contributions to spatial working memory. Neuroscience.

[69] Alves PN (2019). An improved neuroanatomical model of the default-mode network reconciles previous neuroimaging and neuropathological findings. Commun. Biol..

[70] Wang X (2016). Subcomponents and connectivity of the superior longitudinal fasciculus in the human brain. Brain Struct. Funct..

[71] Schmahmann JD (2007). Association fibre pathways of the brain: Parallel observations from diffusion spectrum imaging and autoradiography. Brain.

[72] Wertz CJ (2020). White matter correlates of creative cognition in a normal cohort. Neuroimage.

[73] Brewer JA (2011). Meditation experience is associated with differences in default mode network activity and connectivity. Proc. Natl. Acad. Sci..

[74] Van Den Heuvel M (2008). Microstructural organization of the cingulum tract and the level of default mode functional connectivity. J. Neurosci..

[75] Linden DE (2006). How psychotherapy changes the brain—The contribution of functional neuroimaging. Mol. Psychiatry.

[76] Peng X (2020). Impaired left amygdala resting state functional connectivity in subthreshold depression individuals. Sci. Rep..

[77] Roeckner AR (2021). Neural contributors to trauma resilience: A review of longitudinal neuroimaging studies. Transl. Psychiatry.

[78] Pisner D (2019). The superior longitudinal fasciculus and its functional triple-network mechanisms in brooding. NeuroImage Clin..

[79] Zheng Y (2021). Diffusion property and functional connectivity of superior longitudinal fasciculus underpin human metacognition. Neuropsychologia.

[80] Hwang Y-G (2023). Self-compassion is associated with the superior longitudinal fasciculus in the mirroring network in healthy individuals. Sci. Rep..

[81] Sekiguchi A (2014). White matter microstructural changes as vulnerability factors and acquired signs of post-earthquake distress. PLoS One.

[82] Kim MJ (2019). Microstructural integrity of white matter moderates an association between childhood adversity and adult trait anger. Aggress. Behav..

[83] Martin AJ, Marsh HW (2006). Academic resilience and its psychological and educational correlates: A construct validity approach. Psychol. Sch..

[84] Seligman ME, Csikszentmihalyi M (2000). Positive Psychology: An Introduction.

[85] Davey M (2003). Resilience processes in adolescents: Personality profiles, self-worth, and coping. J. Adolesc. Res..

[86] Riolli L (2002). Resilience in the face of catastrophe: Optimism, personality, and coping in the Kosovo crisis. J. Appl. Soc. Psychol..

[87] Bolsinger J (2018). Neuroimaging correlates of resilience to traumatic events—A comprehensive review. Front. Psychiatry.

[88] Shi L (2019). Recover from the adversity: Functional connectivity basis of psychological resilience. Neuropsychologia.

[89] Connor KM, Davidson JR (2003). Development of a new resilience scale: The Connor–Davidson Resilience Scale (CD-RISC). Depress. Anxiety.

[90] Baek H-S (2010). Reliability and validity of the Korean version of the Connor–Davidson Resilience Scale. Psychiatry Investig..

[91] Folkman S, Lazarus RS (1980). An analysis of coping in a middle-aged community sample. J. Health Soc. Behav..

[92] Folkman S (1986). Appraisal, coping, health status, and psychological symptoms. J. Pers. Soc. Psychol..

[93] Folkman S (1984). Personal control and stress and coping processes: A theoretical analysis. J. Pers. Soc. Psychol..

[94] Kim HS (2021). Psychometrics properties of ways of coping questionnaire-Korean among college students. Korean J. Health Promot..

[95] Marteau TM, Bekker H (1992). The development of a six-item short-form of the state scale of the Spielberger State—Trait Anxiety Inventory (STAI). Br. J. Clin. Psychol..

[96] Spielberger CD, Craighead WE, Weiner IB (2010). The Corsini Encyclopedia of Psychology.

[97] Han DW (2008). Korean State-Trait Anxiety Inventory application study in middle and high school students. Korean Psychol. Assoc..

[98] Beck AT (1988). An inventory for measuring clinical anxiety: Psychometric properties. J. Consult. Clin. Psychol..

[99] Lee H-K (2016). Psychometric properties of the Beck Anxiety Inventory in the community-dwelling sample of Korean adults. Korean J. Clin. Psychol..

[100] Beck AT (1961). An inventory for measuring depression. Arch. Gen. Psychiatry.

[101] Dozois DJ (1998). A psychometric evaluation of the Beck Depression Inventory-II. Psychol. Assess..

[102] Ware J (1993). SF-36 Health Survey: Manual and Interpretation Guide.

[103] Han C-W (2004). Development of the Korean version of Short-Form 36-Item Health Survey: Health related QOL of healthy elderly people and elderly patients in Korea. Tohoku J. Exp. Med..

[104] Ashburner J, Friston KJ (2000). Voxel-based morphometry—The methods. Neuroimage.

[105] Brett, M. *et al.* Region of interest analysis using an SPM toolbox. In *8th International Conference on Functional Mapping of the Human Brain*, 16, 497 (2002).

[106] Hagler DJ (2006). Smoothing and cluster thresholding for cortical surface-based group analysis of fMRI data. Neuroimage.

[107] Schaer M (2008). A surface-based approach to quantify local cortical gyrification. IEEE Trans. Med. Imaging.

[108] Kelly PA (2013). Cortical thickness, surface area, and gyrification abnormalities in children exposed to maltreatment: Neural markers of vulnerability?. Biol. Psychiatry.

[109] Desikan RS (2006). An automated labeling system for subdividing the human cerebral cortex on MRI scans into gyral based regions of interest. Neuroimage.

[110] Fischl B (2004). Automatically parcellating the human cerebral cortex. Cereb. Cortex.

[111] Smith SM (2006). Tract-based spatial statistics: Voxelwise analysis of multi-subject diffusion data. Neuroimage.

[112] Alfaro-Almagro F (2018). Image processing and Quality Control for the first 10,000 brain imaging datasets from UK Biobank. Neuroimage.

[113] Hagler DJ (2006). Smoothing and cluster thresholding for cortical surface-based group analysis of fMRI data. Neuroimage.

[114] Faul F (2009). Statistical power analyses using G* Power 31: Tests for correlation and regression analyses. Behav. Res. Methods.

