# Supplementary material for: Multimodal neural correlates of dispositional resilience among healthy individuals
Source: Sci Rep. 2024 Apr 30;14:9875. doi: 10.1038/s41598-024-60619-0 (PMC11059361; doi:10.1038/s41598-024-60619-0)
Supplement: Supplementary file 1 — Supplementary Information. [file 41598_2024_60619_MOESM1_ESM.docx]

**Supplementary material**

**Supplementary Results**

**The structural neural correlates of the subscales of resilience**

In terms of examining the structural neural correlates of the subscales of the Korean version of the Connor-Davidson Resilience Scale (K-CD-RISC), the gray matter volume (GMV) or cortical thickness (CT) of any brain region did not show any significant correlations with subscale scores among healthy individuals. However, as presented in Supplementary Figure 1, the local gyrification index (LGI) in the superior temporal gyrus (STG) and supramarginal gyrus (SMG) of the left hemisphere was significantly positively correlated with the K-CD-RISC subscale *hardiness* scores among healthy individuals [cluster wise *p*-value (CWP)<0.05]. The LGI in the insula (insula 2^*^) and rostral middle frontal (RMF) gyrus of the left hemisphere was significantly positively correlated with the K-CD-RISC subscale *persistence* scores in healthy individuals (CWP<0.05).

In addition, our study found a significant negative correlation between the scores of the *persistence* subscale of the K-CD-RISC and mean fractional anisotropy (FA) values in three cluster regions (the second branch of the superior longitudinal fasciculus [SLF II 2^*^], inferior longitudinal fasciculus [ILF], and posterior white matter [WM] of the SMG in the right hemisphere; Supplementary Figure 2).

(^*^The number ‘2’ was added to the regions of neural correlates of the subscales to distinguish them from the neural correlates of the K-CD-RISC total scores described in the main manuscript.)

**Correlation analyses between the structural neural correlates of resilience subscales and psychological measures in healthy individuals**

The structural neural correlates of the K-CD-RISC subscale *hardiness* were the LGI of the STG and SMG regions. The LGI of the STG was associated with the Beck Anxiety Inventory (BAI) score (*rho*=-0.347, *p*-value=0.005) and 36-item Short Form Health Survey (SF-36) general health (*rho*=0.391, *p*-value=0.002). The LGI of the SMG was positively correlated with SF-36 subscales, such as physical functioning (*rho*=0.408, *p*-value=0.001) and general health (*rho*=0.355, *p*-value=0.004). In addition, the structural neural correlates of the *persistence* subscale of the K-CD-RISC were the LGI in insula 2 and RMF regions. The LGI of insula 2 was related to the BAI (*rho*=-0.331, *p*-value=0.008) and SF-36 general health subscale (*rho*=0.370, *p*-value=0.003) scores. The LGI of the RMF was positively associated with SF-36 general health subscale scores (*rho*=0.343, *p*-value=0.006).

The mean FA values of the SLF II 2 related to the K-CD-RISC subscale *persistence* scores were significantly negatively correlated with the SF-36 general health (*rho*=-0.339, *p*=0.004) and problem-focused coping style (*rho*=-0.305, *p*-value=0.009) scores among healthy individuals. Furthermore, the lower the mean FA values of the ILF related to the subscale *persistence* scores of the K-CD-RISC, the higher the problem-focused coping style scores (*rho*=-0.294, *p*-value=0.011) in healthy individuals (Supplementary Figure 4).

**Supplementary Discussion**

In the subscale analyses, the LGIs in the STG and SMG of the left hemisphere were significantly positively correlated with the hardiness subscale scores of the K-CD-RISC. The STG is involved in auditory processing, including language, but it has also been implicated as a critical structure in social cognition ^1^. In addition, the SMG is involved in identifying the postures and gestures of other people and is thus a part of the mirror neuron system (MNS) ^2^. Hardiness can be defined as the ability to adapt easily to unexpected changes, combined with a sense of purpose in daily life and personal control over what occurs in one’s life. Hardiness dampens the negative effects of stress and can be a protective factor against illnesses ^3^. Research has shown that hardiness is closely related to resilience, in which biologically MNS can be involved, and that the coping style associated with hardiness is transformational coping, an optimistic style ^4^.

In addition, in the subscale analyses, the LGIs in insular 2 and RMF were significantly positively associated with the persistence subscale scores of the K-CD-RISC. Persistence can be interpreted as the ability to maintain a particular behavior despite stress ^5^. Both the insula and RMF, part of the dorsolateral prefrontal cortex, are involved in emotional regulation and regulation of the inner body state ^6-8^. The insula is known to be involved in expressive suppression, an emotional regulation strategy that requires interoceptive and emotional awareness ^9^. However, one of the functions of RMF is high-level emotion regulation and the sustained effects of cognitive emotion regulation ^10^. Structural changes in the RMF are related to trait resilience ^11^. Taken together, the insula and RMF appear to play a role in persistence. Findings of LGI changes in the insula and RMF related to resilience can be understood in this context.

In our correlation analyses, increased LGI clusters (STG, SMG, insula, and RMF) were significantly correlated with low levels of anxiety and increased quality of life, especially in physical functioning and general health categories. As mentioned above, the STG, SMG, and insula regions are all included in the MNS, and the RMF is known to function in higher-level emotional regulation. Thus, clusters of increased LGI as resilience-related structural neural correlates may play a role in the regulation of emotion, which contributes to a high quality of life perception.

This study showed that persistently healthy individuals had significantly lower FA values in the ILF and DMN-related posterior division of the WM region adjacent to the SMG. Psychological persistence, one of the subscales of dispositional resilience, focuses on trust in one’s instincts, tolerance of negative affect, and strengthening the effects of stress ^12^. The direct mechanisms underlying decreased FA in the ILF and posterior division of the SMG WM region among highly resilient individuals remain unclear. However, the ILF is a ventral associative fiber connecting the default mode network (DMN) and the MNS-related posterior cingulate cortex or precuneus to the middle temporal lobes, which play an important role in vision-specific emotional language processes, such as human face perception ^13^, visual perception ^14^, and language functions ^15^, which are related to social cognition ^16,17^. The SMG is located in the inferior parietal lobe, a typical part of the MNS ^18^. A previous study showed increased functional connectivity within the precuneus-based posterior DMN in patients with maltreated depression ^19^. Taken together, our results suggest that lower FA values in the ILF and posterior division of the WM region adjacent to the SMG might be associated with better social cognition and cognitive-emotional regulation, which is an important capacity for resilience in healthy individuals.

**References**

1 Bigler, E. D. *et al.* Superior temporal gyrus, language function, and autism. *Dev neuropsychol* **31**, 217-238 (2007). https://doi.org:https://doi.org/10.1080/87565640701190841

2 Reed, C. L. & Caselli, R. J. The nature of tactile agnosia: a case study. *Neuropsychologia* **32**, 527-539 (1994). https://doi.org:https://doi.org/10.1016/0028-3932(94)90142-2

3 VandenBos, G. R. *APA dictionary of psychology*. (American Psychological Association, 2007).

4 Maddi, S. R. The personality construct of hardiness: I. Effects on experiencing, coping, and strain. *Consult Psychol J* **51**, 83 (1999). https://doi.org:http://dx.doi.org/10.1037/1061-4087.51.2.83

5 Clark, W., Holling, C. & Jones, D. Towards a structural view of resilience. *IIASA Working Paper* (1975).

6 Phillips, M. L., Drevets, W. C., Rauch, S. L. & Lane, R. Neurobiology of emotion perception I: The neural basis of normal emotion perception. *Biol Psychiatry* **54**, 504-514 (2003). https://doi.org:https://doi.org/10.1016/S0006-3223(03)00168-9

7 Damasio, A. R. *The feeling of what happens: Body and emotion in the making of consciousness*. (Houghton Mifflin Harcourt, 1999).

8 Golkar, A. *et al.* Distinct contributions of the dorsolateral prefrontal and orbitofrontal cortex during emotion regulation. *PloS one* **7**, e48107 (2012).

9 Giuliani, N. R., Drabant, E. M., Bhatnagar, R. & Gross, J. J. Emotion regulation and brain plasticity: expressive suppression use predicts anterior insula volume. *Neuroimage* **58**, 10-15 (2011). https://doi.org:https://doi.org/10.1016/j.neuroimage.2011.06.028

10 Erk, S. *et al.* Acute and sustained effects of cognitive emotion regulation in major depression. *J Neurosci* **30**, 15726-15734 (2010). https://doi.org:https://doi.org/10.1523/JNEUROSCI.1856-10.2010

11 Zilcha‐Mano, S. *et al.* Structural brain features signaling trauma, PTSD, or resilience? A systematic exploration. *Depress Anxiety* (2022). https://doi.org:https://doi.org/10.1002/da.23275

12 Baek, H.-S., Lee, K.-U., Joo, E.-J., Lee, M.-Y. & Choi, K.-S. Reliability and validity of the Korean version of the Connor-Davidson Resilience Scale. *Psychiatry Investig* **7**, 109 (2010). https://doi.org:https://doi.org/10.4306/pi.2010.7.2.109

13 Fox, C. J., Iaria, G. & Barton, J. J. Disconnection in prosopagnosia and face processing. *Cortex.* **44**, 996-1009 (2008). https://doi.org:https://doi.org/10.1016/j.cortex.2008.04.003

14 Ffytche, D. H. & Catani, M. Beyond localization: from hodology to function. *Philos Trans R Soc B Biol Sci.* **360**, 767-779 (2005).

15 Catani, M. & Mesulam, M. The arcuate fasciculus and the disconnection theme in language and aphasia: history and current state. *Cortex.* **44**, 953-961 (2008). https://doi.org:http://dx.doi.org/10.1016/j.cortex.2008.04.002

16 Wang, Y., Metoki, A., Alm, K. H. & Olson, I. R. White matter pathways and social cognition. *Neurosci Biobehav Rev.* **90**, 350-370 (2018). https://doi.org:https://doi.org/10.1016/j.neubiorev.2018.04.015

17 Buckner, R. L., Andrews-Hanna, J. R. & Schacter, D. L. The brain's default network: anatomy, function, and relevance to disease. *Ann N Y Acad Sci.* **1124**, 1-38 (2008). https://doi.org:https://doi.org/10.1196/annals.1440.011

18 Rizzolatti, G. & Craighero, L. The mirror-neuron system. *Annu. Rev. Neurosci.* **27**, 169-192 (2004). https://doi.org:https://doi.org/10.1146/annurev.neuro.27.070203.144230

19 Li, B. *et al.* A treatment-resistant default mode subnetwork in major depression. *Biol Psychiatry* **74**, 48-54 (2013). https://doi.org:https://doi.org/10.1016/j.biopsych.2012.11.007
