# Supplementary material for: Multimodal neural correlates of dispositional resilience among healthy individuals
Source: Sci Rep. 2024 Apr 30;14:9875. doi: 10.1038/s41598-024-60619-0 (PMC11059361; doi:10.1038/s41598-024-60619-0)
Supplement: Supplementary file 2 — Supplementary Figures. [file 41598_2024_60619_MOESM2_ESM.pptx]

## Slide 1
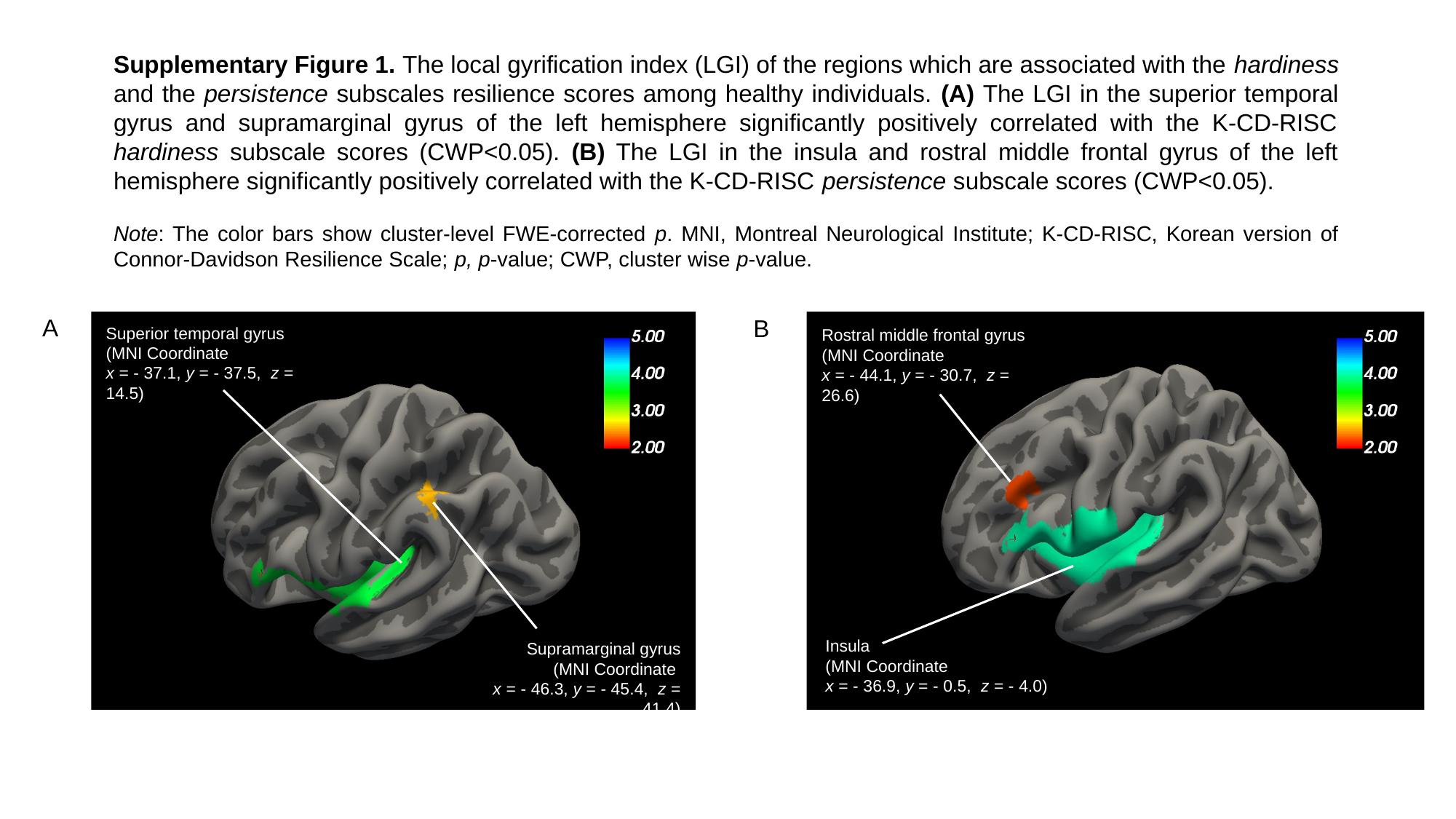

Supplementary Figure 1. The local gyrification index (LGI) of the regions which are associated with the hardiness and the persistence subscales resilience scores among healthy individuals. (A) The LGI in the superior temporal gyrus and supramarginal gyrus of the left hemisphere significantly positively correlated with the K-CD-RISC hardiness subscale scores (CWP<0.05). (B) The LGI in the insula and rostral middle frontal gyrus of the left hemisphere significantly positively correlated with the K-CD-RISC persistence subscale scores (CWP<0.05).
Note: The color bars show cluster-level FWE-corrected p. MNI, Montreal Neurological Institute; K-CD-RISC, Korean version of Connor-Davidson Resilience Scale; p, p-value; CWP, cluster wise p-value.
A
B
Superior temporal gyrus
(MNI Coordinate
x = - 37.1, y = - 37.5, z = 14.5)
Rostral middle frontal gyrus
(MNI Coordinate
x = - 44.1, y = - 30.7, z = 26.6)
S
S
A
P
A
P
Insula
(MNI Coordinate
x = - 36.9, y = - 0.5, z = - 4.0)
Supramarginal gyrus
(MNI Coordinate
x = - 46.3, y = - 45.4, z = 41.4)
I
I

## Slide 2
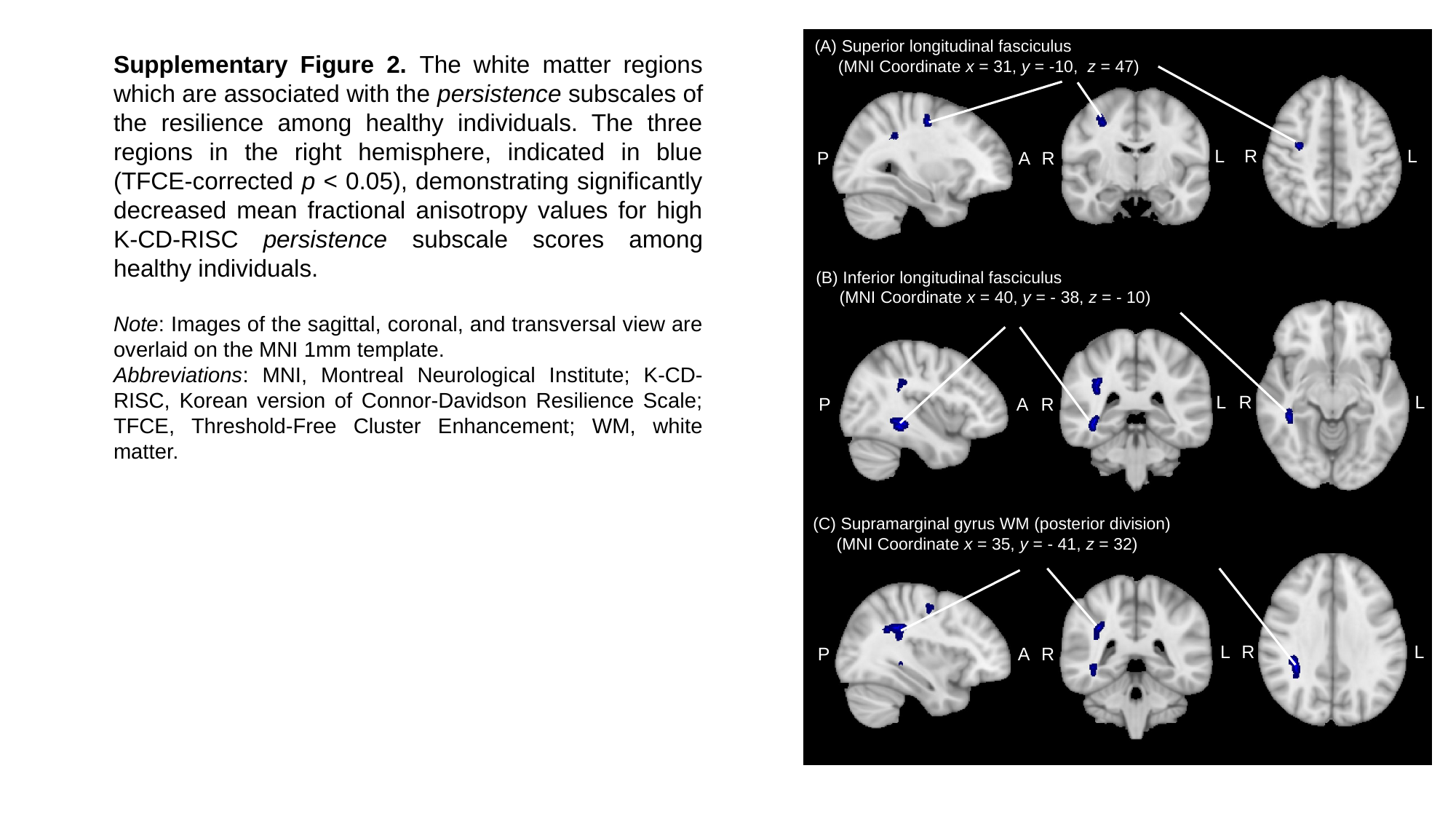

(A) Superior longitudinal fasciculus
 (MNI Coordinate x = 31, y = -10, z = 47)
(B) Inferior longitudinal fasciculus
 (MNI Coordinate x = 40, y = - 38, z = - 10)
(C) Supramarginal gyrus WM (posterior division)
 (MNI Coordinate x = 35, y = - 41, z = 32)
Supplementary Figure 2. The white matter regions which are associated with the persistence subscales of the resilience among healthy individuals. The three regions in the right hemisphere, indicated in blue (TFCE-corrected p < 0.05), demonstrating significantly decreased mean fractional anisotropy values for high K-CD-RISC persistence subscale scores among healthy individuals.
Note: Images of the sagittal, coronal, and transversal view are overlaid on the MNI 1mm template.
Abbreviations: MNI, Montreal Neurological Institute; K-CD-RISC, Korean version of Connor-Davidson Resilience Scale; TFCE, Threshold-Free Cluster Enhancement; WM, white matter.
L
R
L
P
A
R
L
R
L
P
A
R
L
R
L
P
A
R

## Slide 3
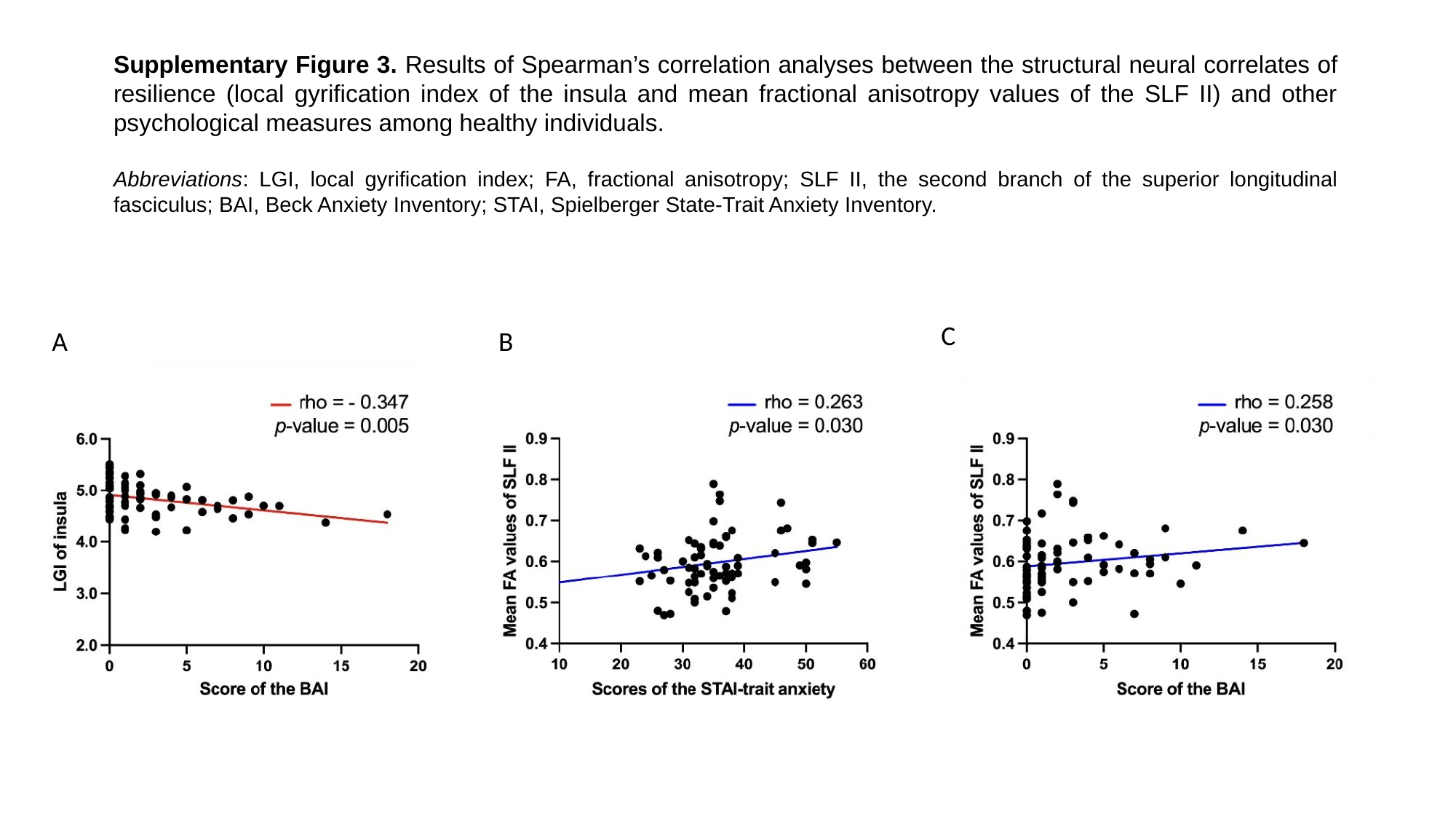

Supplementary Figure 3. Results of Spearman’s correlation analyses between the structural neural correlates of resilience (local gyrification index of the insula and mean fractional anisotropy values of the SLF II) and other psychological measures among healthy individuals.
Abbreviations: LGI, local gyrification index; FA, fractional anisotropy; SLF II, the second branch of the superior longitudinal fasciculus; BAI, Beck Anxiety Inventory; STAI, Spielberger State-Trait Anxiety Inventory.
C
A
B

## Slide 4
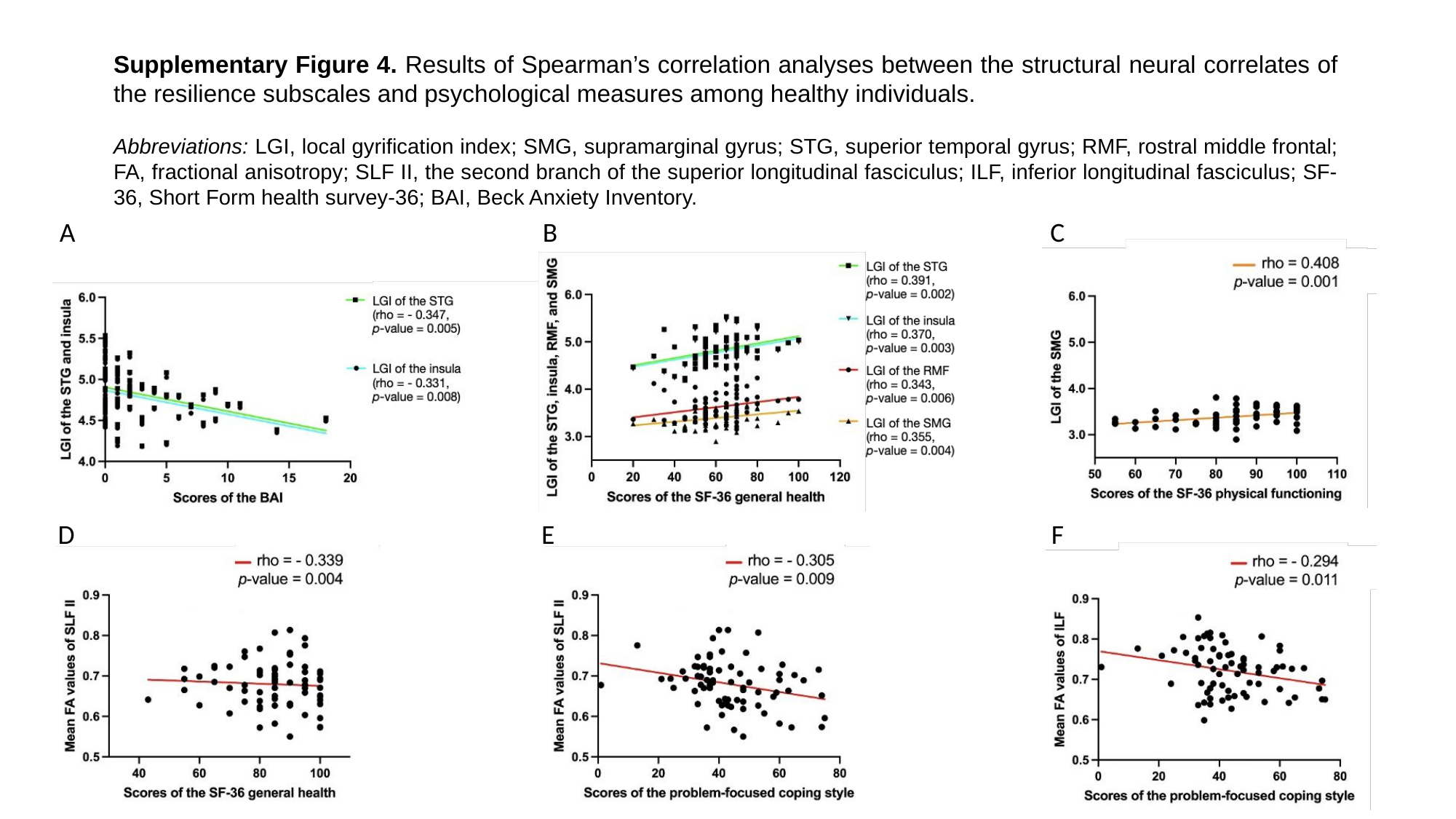

Supplementary Figure 4. Results of Spearman’s correlation analyses between the structural neural correlates of the resilience subscales and psychological measures among healthy individuals.
Abbreviations: LGI, local gyrification index; SMG, supramarginal gyrus; STG, superior temporal gyrus; RMF, rostral middle frontal; FA, fractional anisotropy; SLF II, the second branch of the superior longitudinal fasciculus; ILF, inferior longitudinal fasciculus; SF-36, Short Form health survey-36; BAI, Beck Anxiety Inventory.
A
B
C
D
E
F
